# Non-invasive estimation of glomerular filtration rate (GFR). The Lund model: Simultaneous use of cystatin C- and creatinine-based GFR-prediction equations, clinical data and an internal quality check

**DOI:** 10.3109/00365511003642535

**Published:** 2010-02-22

**Authors:** Anders Grubb

**Affiliations:** ^a^ Department of Clinical Chemistry, University Hospital, Lund, Sweden

**Keywords:** Kidney function tests, estimation of glomerular filtration rate, creatinine, cystatin C, renal failure

## Abstract

Knowledge of glomerular filtration rate (GFR) is required to detect and follow impairment of renal function, to allow correct dosage of drugs cleared by the kidneys, and for the use of nephrotoxic contrast media. Correct determination of GFR requires invasive techniques, which are expensive, slow and not risk-free. Therefore, GFR-prediction equations based solely upon cystatin C or creatinine and anthropometric data or upon cystatin C, creatinine and anthropometric data have been developed. The combined prediction equations display the best diagnostic performance, but in several easily identifiable clinical situations (e.g. abnormal muscle mass, treatment with large doses of glucocorticoids) prediction equations based upon either cystatin C or creatinine are better than the combined equations. In Lund, where cystatin C has been used as a GFR-marker in the clinical routine since 1994, a strategy based upon this knowledge has therefore been developed. This comprises simultaneous use of a cystatin C-based and a creatinine-based GFR-prediction equation. If the GFRs predicted agree, the mean value is used as a reliable GFR-estimate. If the GFRs predicted do not agree, clinical data is evaluated to identify reasons for not using one of the two prediction equations and the GFR predicted by the other one is used. If no reasons for the difference in predicted GFRs are found, an invasive gold standard determination of GFR is performed. If the GFRs predicted agree for a patient, the creatinine value is reliably connected to a specific GFR and can be used to follow changes in GFR of that patient.

## Introduction

Knowledge of glomerular filtration rate (GFR) is required to detect and follow impairment of renal function, to allow correct dosage of drugs cleared by the kidneys and for the use of potentially nephrotoxic radiographic contrast media. GFR in humans cannot be measured directly and invasive techniques based on measuring the plasma or renal clearance rate of injected substances (e.g. inulin, ^51^Cr-EDTA, iohexol, ^125^I-iothalamate, ^99m^Tc-diethylelenetriaminepentaacetic acid) that are exclusively excreted via glomerular filtration are therefore required for the correct measurement of GFR. However, these so called gold standard techniques cannot be generally applied because they are labour-intensive, expensive and not entirely free of risk for the patient. The plasma or serum concentrations of endogenous substances, particularly creatinine, have therefore been used as markers for GFR for almost a century. But it has become evident that the creatinine level alone is far from ideal as a GFR marker because it is significantly influenced not only by GFR but also by muscle mass, diet, gender, age, drugs and tubular secretion [1].

Cystatin C was first suggested as a new marker for GFR in 1979, when it was observed that the plasma level of cystatin C was up to 13 times higher in patients on haemodialysis than in healthy persons [2]. One of the methods developed in 1979 for determination of the cystatin C level in body fluids was enzyme-amplified single radial immunodiffusion [2]. Although this procedure was slow and had a coefficient of variation of 11%, it was useful for identification of cystatin C as a GFR-marker at least as good as creatinine, since the correlation coefficients for the relation between the serum levels of cystatin C and GFR, determined by a gold standard method (plasma clearance of ^51^Cr-EDTA), were somewhat higher than that between creatinine and GFR [3,4]. However, development of automated, rapid and precise methods for determination of the serum or plasma level of cystatin C was required for the use of cystatin C as a marker for GFR in the clinical routine. The first method of this type, a particle-enhanced immunoturbidimetric method, was developed in 1994 [5] and applied for determination of the serum cystatin C levels in a cohort of 51 patients with GFR measured by a gold standard procedure. ROC curve analysis demonstrated that in this cohort of patients, serum cystatin C had a significantly better diagnostic performance than serum creatinine [5]. Since then, several automated, rapid and precise methods for determination of cystatin C have been developed [6–12] and the information on cystatin C as a GFR-marker has substantially increased. Entering ‘cystatin C AND (glomerular OR renal)’ in the search field of www.ncbi.nlm.nih.gov/pubmed in February 2010 generated 1031 hits.

The main advantage of cystatin C compared to creatinine as a GFR-marker is that it is less dependent upon the body composition of a patient than creatinine. For example, while muscle mass strongly influences creatinine, it does not, or only marginally, affects cystatin C [13–15]. Muscle loss of a patient, e.g. by paralysis, immobilization or low mobility, involuntary or voluntary malnutrition (anorexia), will strongly impair the use of creatinine as a GFR-marker, but not that of cystatin C [16,17]. Muscle loss during aging reduces the production of creatinine and therefore impairs the use of creatinine to follow the decline of GFR with age. But the cystatin C production is not strongly influenced by muscle mass and cystatin C will therefore increase with age in parallel with the decrease of GFR and therefore be more useful than creatinine to demonstrate the normal and abnormal decrease in GFR in the elderly [18–28]. The muscle mass of children up to around the age of 18 years varies considerably; therefore, age-specific reference values for creatinine are required, whereas the cystatin C level is virtually constant from 1 year, allowing uniform reference values for cystatin C in children [29–33].

## Creatinine- and cystatin C-based GFR-prediction equations

**Figure 1.**
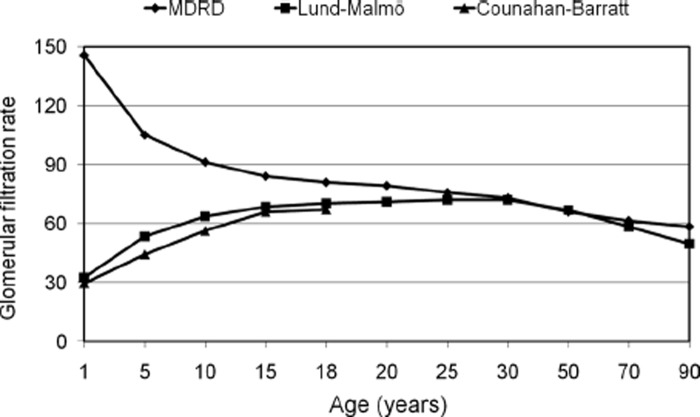
Age-related predictions of glomerular filtration rate (mL/min/1.73 m^2^) at a constant creatinine level of 80 μmol/L for a female using the Lund-Malmö (LM) [44], the MDRD [37] or the Counahan-Barratt equations [43,44]. The mean heights of Swedish children of different ages [63] were used for the Counahan-Barratt equation.

Since creatinine alone has clear drawbacks as a GFR-marker, it is widely considered that it should be replaced by GFR-prediction equations based not only upon creatinine, but also upon anthropometric and/or demographic data such as sex, age and ethnicity, to compensate for the influence of muscle mass on the creatinine level [34]. Most of the well-founded, generally used and recommended creatinine-based GFR-prediction equations, e.g. the MDRD- and CKD-EPI-equations [35–38], implicitly use the mean muscle mass of a person of a specified age, sex and ethnic origin in the population employed to derive the equation, to compensate for the muscle mass influence on the creatinine-level used for prediction of GFR. If a person’s muscle mass deviates from the mean of that of persons of his/her age, sex and ethnic origin in the population, the GFR-prediction equation will not be accurate for that person. This is an important reason for the remaining imprecision in the creatinine-based GFR-prediction equations. It also explains why different creatinine-based equations are required for maximal diagnostic performance in different populations of individuals, as the relation between muscle mass, age, sex and ethnicity differs between different populations. For example, the MDRD-equation generally underestimates the GFR of healthy people by 29% [39] and its application in a Japanese population requires a Japanese-specific coefficient of 0.763 [40]. Nevertheless, in many clinical situations the creatinine-based GFR-prediction equations estimate GFR at least as well as cystatin C alone [41]. One drawback with presently available creatinine-based GFR-prediction equations is that they usually do not work for persons below 18 years of age, for which specialized prediction equations, e.g. those of Schwartz and Counahan-Barratt, have to be used [42,43]. However, recently a creatinine-based GFR-prediction equation, the Lund-Malmö (LM) equation, which works for both adults and children, has been described [44,45] (Figure 1).

Since the level of cystatin C is less dependent upon anthropometric data than that of creatinine, simpler cystatin C-based GFR-prediction equations of the type GFR = A × cystatin C ^–B^ can be used both for adults and children [32,33,46–48]. Although cystatin C generally seems to be significantly less dependent upon anthropometric data than creatinine [49], this must be verified for patient and ethnic groups not yet studied. It should also be considered that whereas cystatin C alone and cystatin C-based GFR-prediction equations are less influenced by variation in muscle mass than creatinine alone and creatinine-based GFR-prediction equations, the usefulness of cystatin C-based prediction equations are impaired in the same way as cystatin C alone by moderate and high doses of glucocorticoids which increase the synthesis of cystatin C [50–54].

A considerable number of creatinine- or cystatin C-based GFR-prediction equations have been described [33–49]. The reasons for the present high number of equations are the use of different calibrators, the use of non-accurate methods for determinations of creatinine or cystatin C, the use of different patient or ethnic populations and the use of different mathematical models to generate the prediction equations. These factors must be carefully considered before a GFR-prediction equation is selected for use in a particular patient population. For example, when a prediction equation, based upon a specific cystatin C calibrator and determination method, is used to estimate GFR from the cystatin C levels produced using another cystatin C calibrator and determination method, large errors in the resulting GFR-estimates may result, even for similar patient populations. One way of reducing the problems associated with the selection of a suitable GFR-prediction equation is to produce international calibrators for creatinine and cystatin C and use them, not only to secure the use of standardized calibrators in different methods, but also to develop and secure accurate methods for both cytatin C and creatinine. The use of validated international calibrators and accurate methods for determination of creatinine and cystatin C will decrease the number of validated equations and simplify the selection of an equation suitable for a specific patient population. An international calibrator for creatinine is already available [37] and work is in progress to produce one for cystatin C [55].

Although some creatinine- or cystatin C-based GFR-prediction equations produce estimated GFR-values 80–85% of which are between ± 30% of GFR measured by invasive gold standard methods in some studies, the highest percentages of estimated GFR-values between ± 30% of measured GFR-values are obtained using GFR-prediction equations based upon both cystatin C and creatinine [41,49,56–60]. Such equations might produce estimated GFR- values 90–91% of which are between ± 30% of GFR measured by gold standard methods [41,59]. The imprecision of all gold standard procedures means that even if a gold standard procedure is performed twice within a short interval on patients with stable kidney function, less than 100% of the second determination will be within ± 30% of the first. Thus, a GFR-prediction equation producing GFR-values 90–91% of which are within ± 30% of GFR measured by gold standard methods is close to what is theoretically attainable. It should, in addition, be considered that in evaluations of GFR-prediction equations, it is generally, but erroneously, assumed that the imprecision of the gold standard procedure used is 0%. This means that the calculated percentage of estimated GFR-values between ± 30% of ‘true’ GFR is decreased because of the actual imprecision of the gold standard procedure.

## The Lund model: Simultaneous use of cystatin C- and creatinine-based GFR-prediction equations, clinical data and an internal quality check

Although GFR-prediction equations based upon both cystatin C and creatinine clearly seem to have better diagnostic performance than prediction equations based upon only one of these GFR-markers, such combined equations are not optimal in all clinical situations. To give some examples: in a patient suffering from paralysis with very low muscle mass, the combined prediction equation will be less reliable than a prediction equation based on cystatin C alone; in a patient treated with high doses of glucocorticoids, the combined prediction equation will be less reliable than a prediction equation using creatinine alone and anthropometric data. Thus, a strategy for GFR-estimation based upon automatic use of a combined prediction equation using both creatinine and cystatin C would consequentially have worse diagnostic performance than a strategy in which both a cystatin C-based and a creatinine-based prediction equation are used, concomitantly taking clinical data into account.

In Lund, where cystatin C has been available for clinical use since 1994 [5], the following strategy for estimation of GFR has been developed [61]. It is based on three sources of information: the plasma levels of cystatin C and creatinine, respectively, as well as knowledge of the clinical context. Age and gender of the patient is always available, since they can be inferred from the unique identity number (Swedish personal registration number) used to identify all patients. Relative GFR (ml/min/1.73m^2^) is estimated both by a prediction equation based upon cystatin C alone as well as by a prediction equation based upon creatinine alone and anthropometric data. The two estimates are compared and, if they are in agreement within specified limits, the arithmetic mean of the two estimates is used. Diagnostically the arithmetic mean of the two estimates has been shown to perform at least as well as more complex ways of combining the two estimates [59]. The specified limits for agreement between the two estimates can either be applied automatically or the physician can stipulate what level of agreement is required for the specific patient under study. A higher degree of agreement and thus accuracy is required when the estimated GFR is employed for dosing of medication with potential adverse side effects, than for evaluating the level of GFR in a patient. If the two estimates agree, the arithmetic mean is a very reliable estimate of GFR. In fact, during the 15 years we have been using cystatin C in parallel with creatinine as a marker for GFR, we have had about ten cases in which the GFR-estimates based upon cystatin C and creatinine agreed, but disagreed with GFR measured by our invasive gold standard procedure (plasma clearance of iohexol). In all the ten cases, it turned out that the difference was due to technical problems in executing the gold standard procedure.

Therefore in practice, we consider concordant cystatin C and creatinine-based estimates of GFR to be at least as reliable as GFR measured by invasive gold standard procedures. It has previously been shown that the practical execution of gold standard invasive determinations of GFR is not always reliable [62].

If the GFR-estimate based upon cystatin C only does not agree with that based upon creatinine, the clinical situation is considered, e.g. concerning the presence of an abnormal muscle mass or use of high doses of glucocorticoids. When obvious reasons for not using either the cystatin C- or the creatinine-based estimate are found, only the estimate based upon the other, appropriate, prediction equation is used.

If no obvious reasons for a discrepancy between the GFR-estimate based upon cystatin C alone and upon creatinine alone, respectively, can be found, GFR is measured by an invasive gold standard procedure.

When the GFR of a patient has been estimated according to this strategy, changes in GFR can safely be monitored by determination of creatinine, since this strategy connects a reliable GFR-value to the creatinine level of that particular patient. But if the muscle mass of the patient significantly changes, the strategy involving two GFR-estimates has to be repeated.

The strategy outlined above does not require any particular cystatin C-based or creatinine-based prediction equation to be used. Characterization of the population served by a hospital may be required to select the best prediction equations for that hospital. In Lund we have chosen a cystatin C-based equation working for both children and adults [33] and a creatinine-based equation (the LM-equation, Figure 1) that also works for both adults and children [44,45] thus allowing a simpler implementation of the strategy. The strategy is described at www.egfr.se and this site can also be used to implement it and to calculate absolute GFR from relative GFR, which might be required, e.g. for dosing of medicines cleared by the kidneys.
